# Refining the provider payment system of India’s government-funded health insurance programme: an econometric analysis

**DOI:** 10.1136/bmjopen-2023-076155

**Published:** 2023-10-19

**Authors:** Shankar Prinja, Pankaj Bahuguna, Maninder Pal Singh, Lorna Guinness, Aarti Goyal, Vipul Aggarwal

**Affiliations:** 1 Department of Community Medicine and School of Public Health, Postgraduate Institute of Medical Education and Research, Chandigarh, India; 2 Health Economics and Health Technology Assessment (HEHTA), University of Glasgow, Glasgow, UK; 3 Department of Global Health and Development, London School of Hygiene & Tropical Medicine, London, UK; 4 Centre for Global Development, London, UK; 5 Government of India, National Health Authority, New Delhi, India

**Keywords:** HEALTH ECONOMICS, Health policy, PUBLIC HEALTH

## Abstract

**Abstract:**

**Objectives:**

Reimbursement rates in national health insurance schemes are frequently weighted to account for differences in the costs of service provision. To determine weights for a differential case-based payment system under India’s publicly financed national health insurance scheme, the Ayushman Bharat Pradhan Mantri Jan Arogya Yojana (PM-JAY), by exploring and quantifying the influence of supply-side factors on the costs of inpatient admissions and surgical procedures.

**Design:**

Exploratory analysis using regression-based cost function on data from a multisite health facility costing study—the Cost of Health Services in India (CHSI) Study.

**Setting:**

The CHSI Study sample included 11 public sector tertiary care hospitals, 27 public sector district hospitals providing secondary care and 16 private hospitals, from 11 Indian states.

**Participants:**

521 sites from 57 healthcare facilities in 11 states of India.

**Interventions:**

Medical and surgical packages of PM-JAY.

**Primary and secondary outcome measures:**

The cost per bed-day and cost per surgical procedure were regressed against a range of factors to be considered as weights including hospital location, presence of a teaching function and ownership. In addition, capacity utilisation, number of beds, specialist mix, state gross domestic product, State Health Index ranking and volume of patients across the sample were included as variables in the models. Given the skewed data, cost variables were log-transformed for some models.

**Results:**

The estimated mean costs per inpatient bed-day and per procedure were 2307 and 10 686 Indian rupees, respectively. Teaching status, annual hospitalisation, bed size, location of hospital and average length of hospitalisation significantly determine the inpatient bed-day cost, while location of hospital and teaching status determine the procedure costs. Cost per bed-day of teaching hospitals was 38–143.4% higher than in non-teaching hospitals. Similarly, cost per bed-day was 1.3–89.7% higher in tier 1 cities, and 19.5–77.3% higher in tier 2 cities relative to tier 3 cities, respectively. Finally, cost per surgical procedure was higher by 10.6–144.6% in teaching hospitals than non-teaching hospitals; 12.9–171.7% higher in tier 1 cities; and 33.4–140.9% higher in tier 2 cities compared with tier 3 cities, respectively.

**Conclusion:**

Our study findings support and validate the recently introduced differential provider payment system under the PM-JAY. While our results are indicative of heterogeneity in hospital costs, other considerations of how these weights will affect coverage, quality, cost containment, as well as create incentives and disincentives for provider and consumer behaviour, and integrate with existing price mark-ups for other factors, should be considered to determine the future revisions in the differential pricing scheme.

**STRENGTHS AND LIMITATIONS OF THIS STUDY**

- The major strength of analysis is the use of a unique nationally representative of data for India from both public and private hospitals, providing both secondary and tertiary care, located in 11 different states.
- The differences in cost are at the level of individual cost centres that are the key drivers of the reimbursement rates, not the cost of unit of reimbursement—the health benefit package.
- The sample size is relatively small in the context of India and not fully comprehensive: there is a need for further health facility cost data in India in particular from private providers at the specialty and patient level.
- The proposed weights generated through this study are indicative and should be used along with expert consultations.

## Introduction

 Setting an appropriate provider payment system is an important function for a strategic purchaser of healthcare services in any country.^1^ A provider payment system comprises two key aspects: first, what method is used to pay providers, and second, how much is paid for each service delivered. The final payment system can determine how providers engage with the system as well as the overall coverage of health services. For example, the method of payment in terms of either a fee-for-service or capitation or global budget or case-based payment, including diagnostic-related groups (DRG), sets the incentives for the access and coverage of services, cost containment, responsiveness and quality.^2^ At the same time, the rate of payment or the tariff, under any of these methods, should on one hand reflect the cost of resources used to provide services while also incentivising the provision of intended services.^3^

India’s national publicly financed insurance scheme, the Ayushman Bharat Pradhan Mantri Jan Arogya Yojana (PM-JAY), uses a system of case-based payment to reimburse providers for a set of 1574 medical and surgical packages listed under its health benefits scheme.^4^ The rates have been set based on evidence drawn from a nationally representative costing study, which is considered by a wide stakeholder group comprising of clinicians, hospital associations, health economists, development partners and industry representatives.^5 6^ There is a single national reference price for a given package, with mark-ups for a teaching hospital (10%), and for a hospital that has a quality accreditation (10–15%) by the national board.^7^ While there is no denying the logic of giving incentives for and covering the costs of production of more workforce (teaching hospital), higher quality (accreditation), as well as achieving other social policy objectives, there is a need to empirically determine and validate the appropriate level of the mark-up.

While a single national reference price for each package is set under PM-JAY, heterogeneity in health services cost is well established globally.^8 9^ India is a good example of this with significant differences in the healthcare costs by the nature of provider (public or private), type of hospital (secondary or tertiary care), city (tier 1, 2 or 3 city) and state where the hospital is located, and specialist mix within the hospital.^10^,^14^ Previous studies have also shown a seven-time gradient in the cost of hospitalisation within the public sector district hospital from 535 to 3670 Indian rupees in medicine and dermatology, respectively.^13^ If prices do not address these differences in supply-side hospital characteristics, healthcare providers and hospitals, which claim to incur a higher cost of providing care, can become dissatisfied.^15^ This can affect the willingness of hospitals to get empanelled under the scheme and provide care to those who are enrolled.^15^

Price adjustments can help in creating a differential case-based payment system that is sensitive to the hospital characteristics likely to influence the cost of providing healthcare. Examples of such price adjustments can be found in several countries. In the UK, Thailand and Australia, prices are adjusted for providers in different geographical locations to account for differences in local prices and catchment populations.^16^ In Australia, using the DRG-related patient cost data, five sets of cost adjustments are considered for the differential prices: paediatric versus adult population; psychiatry specialty age adjustment; adjustment for greater needs of the indigenous population; patient residential remoteness; and price adjustments for recurrent procedures such as radiotherapy and dialysis. Similarly, in the German DRG system, relative weights are used to account for the variation in resource use for acute care, specialised care or highly specialised care.^17^ Weights for these adjustments are based on evidence drawn from cost analyses of national samples (or census) of participating facilities.^3 18^ Using a strong evidence base to generate such weights provides a transparent way to inform policy that is better able to reflect actual practice, can be defended by the purchaser and set the right incentives for the providers.

In this paper, we present an analysis of the cost data from the first nationally representative hospital costing study—the Cost of Health Services in India (CHSI) Study, to determine supply-side weights which could be used to develop a differential case-based payment system in India, under the PM-JAY.

## Methods

### Selection of factors for price weights

The choice of factors to determine weights was guided by policy need and data availability. Factors considered for weights fall into two categories. First, the supply-side characteristics of hospitals influence the cost of delivering services. These include variations in salaries, types of healthcare providers, rental for space, the specialist mix of the health workforce, scale of service delivery, level of specialisation in service delivery and adherence to quality standards.

Costs and therefore prices, such as salaries and rentals, are in turn determined by geographical factors. For example, the city where a hospital is located determines the cost of several inputs and processes—including the land.^19^ Similarly, the salary paid to a doctor with equivalent qualifications and experience varies with the remoteness of a city where the hospital is located. To capture these differences, we used the classification of the city—tier 1, 2 or 3, where tier 1 is known to be the most expensive and tier 3 the least expensive. The tier-type classification of Indian cities was introduced by seventh pay commission to account for differences in cost of living across the cities and to pay the allowances accordingly.^20^ From a policy perspective, such a factor is advantageous as it is less subject to any gaming by providers. Other supply-side factors considered in our analysis were the size of the hospital (bed strength), level of specialisation of the hospital (secondary vs tertiary), whether the hospital has a teaching and academic role, and the specialist mix of healthcare providers. Table 1 defines the variables considered for the analysis and the hypothesised direction of the associated incentive if used as a weight.

**Table 1:** Choice of variables for a cost function determining weights for reimbursement rates

Concept	Variable used in the model*	Definition	Rationale for inclusion in the model	Mean/percentage of sampleInpatient admissionsN=371	Mean/percentage of sampleSurgical proceduresN=256
Unit cost	Cost per bed-day/procedure	Cost of an inpatient bed-day in INR (2020 prices) across the sampled specialties; mean cost of surgical procedure in INR (2020) prices across the sampled specialties	Dependent variable	2307	10 686
Specialty size	As per definition	Number of beds	Higher prices for smaller hospitals whose capacity limits the benefits of economies of scale	23.6	33.8
Volume of services†	Number of admissions/number of procedures	Number of inpatient admissions per year (inpatient analysis)/number of procedures	Scale of activity	3435	8423
Teaching vs non-teaching (dummy variable)	Teaching facilities	Specialties that have either a teaching function (ie, training doctors or other medical staff) or are a non-teaching facility. Teaching facilities are all public tertiary in the sample, and that non-teaching facilities are a mix of public secondary and private facilities	Higher prices for teaching hospitals have the potential to reward quality and compensate for teaching time	12.9%	18.4%
	Non-teaching facilities			87.1%	81.6%
Location (dummy variable)	Tier 1	Tier classification: city tier classification is based on population size and used by the Indian National Pay Commission as indicator of cost of living for establishing government employee allowances such as house rent allowance (also a proxy indicator of variations in the cost of living or prices)	Higher prices for high tier cities to compensate for a higher cost of living	7.8%	9.8%
	Tier 2			28%	26.2%
	Tier 3			64.2%	64.1%
	State GSDP per capita† (INR)	Per capita GSDP given by the Reserve Bank of India^44^	Higher GSDP likely to result in higher costs	131 914	131 914
	State Health Index†	Composite index to assess the performance of states using indicators of health outcomes, governance and information and key inputs/processes. Index is on a scale of 1–100^25^	Differences in health system performance lead to differences in cost	59	59
Technical efficiency†	Bed occupancy rate (capacity utilisation)	Ratio of occupied bed-days to total bed-days available with specialty/facility)	Scale inefficiencies, present across a sample (cost centres), result in variations in unit costs	0.99	1.03
	Specialist–paramedic ratio	Ratio of number of specialists to number of paramedics staff in a specialty	Facilities with a higher ratio are likely to have higher unit costs	0.38	0.46
	Doctor–paramedic ratio	Ratio of number of doctors to number of paramedics staff in a specialty	Facilities with a higher ratio are likely to have higher unit costs	0.37	0.33

* All variables were obtained from the CHSI data unless otherwise specified.

† Variables not considered for price adjustments as impractical or not considered appropriate for compensation (provides the wrong kind of incentive) but are likely to be a key determinant of costs that needs to be accounted for in the model.

CHSI, Cost of Health Services in India; GSDP, gross state domestic product; INR, Indian rupee.

Demand-side patient characteristics, including case-mix and severity, also influence the cost of care. Case-mix is partly accounted for under the current reimbursement rates as the rates are set at the procedure or disease condition level. The further weighting of case-mix that accounts for severity and age should be assessed through patient-level data which are currently unavailable. Although supply-side weights can account for some degree of a facility’s patient profile through the factors of type of ownership and level of specialisation of the hospital,^21^ this analysis focuses on supply-side weights to complement any future work on patient characteristics.

Cost variations are also derived from differences in the health system as well as technical efficiency. In particular, healthcare financing patterns and infrastructure vary across the Indian states.^22^ The analysis controlled for these health system differences at the state level using the state-level gross domestic product (GDP) per capita and State Health Index (see table 1). Price adjustment also needs to avoid rewarding provider inefficiencies. We included variables to control for differences in technical efficiency in the form of capacity utilisation (ratio of occupied bed-days to total bed-days available with specialty/facility) and skills mix (ratio of more specialist staff to less specialist staff). Finally, the scale was also accounted for using the hospital bed strength and volume of services (table 1).^23^

### Data source

We used the CHSI Study data for the present analysis.^5 6^ This comprises economic provider cost data collected from the set of public and private hospitals in 11 Indian states, using a mix of top-down and bottom-up costing methods.^5 24^ The sample included 11 public sector tertiary care hospitals which also include teaching component, 27 public sector district hospitals providing secondary care and 16 private hospitals. The multistage selection strategy for choosing the sample of hospitals is explained elsewhere.^5 6^ The CHSI data were analysed to estimate the unit costs of service delivery at each of the cost centre including outpatient visit, inpatient bed-day, intensive care and surgical procedures (in the case of surgical specialties). The data were collected between 2018 and 2020 and reported as annual costs in 2020 prices.

A given specialty in a hospital was chosen as the unit of analysis for the present study. Data were analysed for 9, 18 and 19 specialty units or departments from public sector tertiary hospitals, public sector secondary hospitals and private hospitals, respectively. Of these, 15 specialty units/departments belonged to hospitals located in tier 1 cities, while 18 and 20 specialties were representative of hospitals in tier 2 and 3 cities, respectively. For each of the specialties, the dataset comprised of variables for hospital characteristics, cost weight variables and control variables, as well as the unit costs for each of the cost centres.^25 26^

### Patient and public involvement

Neither patients nor the public were involved in the design, conduct, reporting or dissemination plans of our research.

### Data analysis

The reimbursement rate under PM-JAY comprises the hospitalisation cost, the procedure cost, pre-admission consultation and diagnostic work-up, as well as the cost of 15-days of medication following discharge. We analysed the CHSI phase I dataset which has shown that on average across all surgical packages of care, 60% of the total cost is derived from the procedure alone.^6 27^ As a result, we focused on the inpatient and procedure cost centres to determine weights of medical and surgical packages, respectively.

#### Model development

We used regression-based statistical methods with the aim to explore the determinants of cost variations by developing an average cost function.^8 28^ We developed a model similar to the WHO-CHOICE cost function for predicting unit costs and previous work done in the Indian context.^8 28^ An ordinary least square method was used for estimation. The estimated coefficients were tested for significance. The regression-based coefficients are used to suggest the weights.

#### Model specification

The model regresses the unit cost as a dependent variable against the set of explanatory variables. Suitable transformation of the explanatory variables was undertaken for use in the models. We used the log form of continuous variables to address skewness in the data. Nominal variables were transformed into dummy variables. These included teaching versus non-teaching hospitals, and city tier classification for hospital location. The unstandardised coefficient interpreted according to the standard recommended methodology which is appropriate when multiple linear regression is undertaken in a log-log fashion, that is, the dependent variable and independent variables were log-transformed. The formula used for interpretation of estimated effect of predictor variables is (e^β^−1)×100%. Models were run with and without outliers.^29^

#### Model statistics and selection

In general, a regression-based average cost function serves two purposes, one to predict the outcome of interest and second to explore the determinants that are significantly associated with the outcome of interest, compared with relative categories within a factor or other determinants. The adjusted r^2^ or coefficient of determination is one of the key measures to assess the best-fit models when we are primarily interested in predictive functions. However, important conclusions can be drawn for independent factors which significantly determine the outcome of interest, even when the model r^2^ is low.^30^

The strength of the relationship between the determinants and unit cost was assessed based on the magnitude of regression coefficients and their statistical significance. Tolerance and variation inflation factor (VIF) measures were computed to examine the presence of multicollinearity in the model. The cut-off value of tolerance <0.1 and VIF >10 indicated the presence of significant multicollinearity in the model.^31^

## Results

Overall, we used cost information from 521 hospital departments or specialty units. More than half of the inpatient units belonged to district hospitals (53.9%) and hospitals in tier 3 cities (64.2%). The distribution of surgical procedure units followed a similar pattern (table 1).

The estimated mean costs per inpatient bed-day and per procedure were 2307 (SD 4982) and 10 686 (14 921) Indian rupees, respectively. While the inpatient bed-day cost was higher in specialties within private hospitals, the procedure cost was highest, on average, in the public tertiary hospitals. The mean cost per procedure was higher among specialties in hospitals located in tier 1 cities (table 2). In view of the skewed nature of cost data, median estimates were also reported. Though there were considerable differences in the mean and median estimates, both followed the same trend across the strata of the different hospital characteristics (table 2).

**Table 2:** Unit cost estimates (Indian rupees) per inpatient bed-day and procedure

Parameters	Cost per bed-day inpatient (Indian rupees)				Cost per procedure (Indian rupees)			
	N	Mean	SD	Median	N	Mean	SD	Median
Overall	256	2307	4982	1314	371	10 686	14 921	6951
Teaching	47	2175	2981	995	48	16 077	21 455	10 452
Non-teaching	209	2327	5218	1360	323	9474	12 772	6260
Tier 1	25	2395	1781	1955	29	20 021	28 217	11 656
Tier 2	67	4023	8756	2605	104	14 289	18 367	10 826
Tier 3	164	1546	1785	966	238	7792	8207	5404
Public	177	1684	2125	856	248	9516	13 217	6035
Private	79	3564	7985	1882	123	13 308	17 990	9199

### Cost function

The final models are presented in tables3  4. The best-fit models were identified as models run on data with outliers for inpatient bed-day and procedure cost. The results of the other models (without outliers for inpatient bed-day and procedure cost) are reported in the online supplemental tables 1 and 2. The final model indicates that the teaching status, bed size, annual inpatient admissions, average length of hospitalisation and location of hospital significantly determine the cost of an inpatient bed-day (table 3). Teaching hospitals incur a bed-day cost from 38% to 143% higher than non-teaching hospitals. Similarly, hospitals located in tier 1 and tier 2 cities have higher costs compared with hospitals located in tier 3 cities, ranging from 1.3% to 89.7%, and 19.5% to 77.3%, respectively.

**Table 3:** Results of unit cost function: inpatient bed-day

Variable	Standardised β	Unstandardised β	SE β	95% CI	
				Lower	Upper
Annual inpatient admissions	−0.955	−0.730***	0.042	−0.813	−0.648
Number of inpatient beds	0.404	0.378***	0.060	0.259	0.496
Average length of stay	−0.379	−0.745***	0.086	−0.915	−0.574
Location (reference: tier 3 city)					
Tier 1	0.084	0.327*	0.159	0.013	0.640
Tier 2	0.158	0.375***	0.100	0.178	0.573
Teaching status (reference: non-teaching hospitals)					
Teaching hospitals	0.206	0.606***	0.144	0.322	0.890
(Constant)		12.403***	1.106	10.223	14.582
R^2^	0.7225				
Adjusted R^2^	0.7115				

Further variables included in the model: doctor–paramedic ratio, specialist–paramedic ratio, absolute GSDP and State Health Index.

**$** Unit cost, inpatient beds, average length of stay and annual admissions are log-transformed.

*P<0.05; **p<0.01; ***p<0.001.

GSDP, gross state domestic product.

**Table 4:** Results of unit cost function: procedure

Variable	Standardised β	Unstandardised β	SE β	95% CI	
				Lower	Upper
Annual procedures	−0.314	−0.154***	0.042	−0.238	−0.070
Bed occupancy	−0.069	−0.057	0.062	−0.180	0.066
Specialty	0.148	0.515	0.270	−0.019	1.049
Location (reference: tier 3 city)					
Tier 1	0.210	0.560*	0.222	0.121	1.00
Tier 2	0.311	0.584***	0.150	0.288	0.879
Teaching status (reference: non-teaching hospitals)					
Teaching hospitals	0.256	0.498*	0.201	0.101	0.894
(Constant)		9.588**	1.600	6.426	12.749
R^2^	0.3243				
Adjusted R^2^	0.2832				

Further variables included in the model: doctor–paramedic ratio, specialist–paramedic ratio, absolute GSDP, annual operation theatre surgical procedures and State Health Index.

Public and private classification variable not included in cost functions as not interested in weights by ‘ownership of hospital’.

**$** Unit cost, inpatient beds, bed occupancy, annual admissions and absolute GSDP are log-transformed.

*P<0.05; **p<0.01; ***p<0.001.

GSDP, gross state domestic product.

The surgical procedure model indicates that annual procedures conducted within the specialty, teaching status and location of hospital were significantly associated with the unit cost per procedure. The costs of surgical procedures in teaching hospitals are higher by 10.6–144.6% than non-teaching hospitals. Similarly, the costs of surgical procedures for hospitals located in tier 1 are higher compared with hospitals located in tier 3 cities by 12.9–171.7%, while tier 2 city hospitals are higher by 33.4–140.9% (table 4).

## Discussion

This paper presents the first study from India to statistically explore the heterogeneity in the cost of hospital care services across different types of facilities and geographical locations, with a sample size sufficient to identify a quantitative relationship. This unique analysis provides the opportunity to propose a system for determining weights to inform the reimbursement rates for India’s national health insurance programme—PM-JAY. Overall, we found that the teaching status (teaching and non-teaching hospitals), location of hospital (tier 1, tier 2 and tier 3 cities), size of hospital, annual hospitalisation (or procedures) and average length of hospitalisation significantly determine the cost of hospital admission. In the case of surgical procedures, the number of procedures, teaching status (teaching and non-teaching hospitals) and location of hospital (tier 1, tier 2 and tier 3 cities) were found to be significantly associated with the unit cost.

Rate setting in case-based reimbursement schemes uses information on the base rate for a particular service or service group that is then weighted according to strategic purposes including case-mix, severity, hospital characteristics and other factors to meet certain social policy objectives. The social policy adjuster serves the purpose of addressing issues such as equity, and in some cases, the budgetary limits.^18 32^ The base rate sets the norm and to avoid embedding inefficiencies or substandard services, should be based on efficient and quality service provision.^33^ Price adjustments can then help address and reimburse for acceptable variations from that norm. Multisite facility costing studies enable policymakers to estimate the base rate as well as politically acceptable variations related to issues of case-mix, equity, local costs and prices and other cost drivers. Countries such as Australia, Germany and the UK rely on these types of studies to generate both the base rate and weights for their reimbursement schemes.^34^,^38^ Our study demonstrates that such an approach can be used in India with the first nationally representative multisite cost estimates.

Our study uses a statistical approach to generate the estimated weights. The approach follows the cost function modelling from the international literature, the majority of which builds on economic theory to predict the costs of service delivery or understand the factors which contribute to heterogeneity in these costs. Factors related to the intensity of care (average length of stay (ALOS); the number of surgeries/outpatient visits/inpatient admissions; nature of care provided, that is, chronic vs rehabilitative, type of specialised care, etc), hospital characteristics (number of beds; teaching vs non-teaching hospital; level of care; ownership of the hospital, capacity utilisation, etc) and other characteristics (geographical factors, GDP, etc) have been reported as determinants of costs in the previous studies.^89 19 28 39^,^41^ Further, a global multicountry study found that ownership of hospitals, teaching status of hospitals, GDP of country, ALOS, bed occupancy rate and number of admissions were significant predictors of cost per bed-day hospitalisation.^8^ An Indian study identified that hospital bed size, annual number of hospital admissions and level of care predominantly predicted the unit cost of hospitalisation at the district hospital level.^42^

Consistent with these findings, our study found that the number of beds, ALOS, annual number of hospital admissions and teaching status of hospitals were significantly associated with heterogeneity in unit costs per bed-day hospitalisation while controlling for state-level variations in financing and system characteristics. In theory, the cost gradients generated from the regression analysis can be used for weighting where there is sufficient justification for differential reimbursement. For example, cost of service provision may also vary according to the location of the hospital as found in the present study.

Under PM-JAY, there is a provision of incentives for hospitals for different supply-side factors, for example, there is an additional incentive in prices to the teaching hospitals, hospitals in tier 1 cities and in aspirational districts of 10% of the standard reimbursement rate. Our findings validate these as well as further incentives to teaching hospitals and in particular hospitals located in tier 1 and tier 2 cities as compared with tier 3 cities for inpatient care.

The cost weights generated through our analysis suggest prices could be set at a much higher level if the goal is to cover the full cost difference. However, the base rate cost of the district hospital does not necessarily reflect good-quality care. If facilities are under-resourced coupled with high volumes of admissions, average costs will be lower than the average cost of efficient high-quality care. In India, district hospitals are known to operate below best practice norms set by the government. A recent health facility survey found the ratio of doctor in position to the government set norms being 0.86 across all states, and that over 25% of sampled district hospitals were operating with bed occupancy above the recommended level of 90% (https://www.niti.gov.in/sites/default/files/2021-10/District_Hospital_Report_for_digital_publication.pdf). This difference from the norms is less pronounced in tertiary care—within our sample, there are on average 25 doctors per bed at district hospital level compared with 11 doctors per bed at tertiary level and bed occupancy of 123% for district hospitals and 104% in tertiary care. The difference between the cost of care provision at a district and a tertiary hospital care is therefore likely to be narrower than represented in our data and price weights informed by the analysis should be adjusted downwards accordingly. The findings also suggest that inpatient care at tier 1 and tier 2 facilities is more costly than at tier 3 hospitals. Similarly, for procedures, tier 1 and tier 2 cities were found to have significantly higher costs as compared with tier 3 cities.

Any review of the weights incorporating the findings would also need to take into account the study limitations. The sample is relatively small given the number of healthcare providers in India and their heterogeneity and as a result is not comprehensive, for example, very large (more than 250 beds) private hospitals located in metro cities were not included. However, the CHSI Study was designed to be nationally representative covering 11 Indian states, and included all types and levels of hospitals, as well as all the major specialties and services, and to account for factors that are likely to influence the cost of care as far as possible. Second, it is not known if the average cost of care within the sample reflects the provision of efficient; good-quality services have not been determined. However, controlling for known drivers of efficiency, the coefficients in the regression are a likely representation of the difference in costs between the different samples. A further limitation relates to the cost data which take a provider perspective, excluding the costs borne by patients which might further vary from location to location and may lead to an underestimate of the cost for specialties with high patient costs. In addition, subanalysis by specialty was not possible, due to the sample size, nor does the procedure or bed-day cost represent the full cost of the health benefit package. As a result, it was not possible to develop a model to help validate the difference in reimbursement rates between packages and it may be that additional factors need to be considered in understanding heterogeneity in health benefit package costs. Nevertheless, the analysis provides a strong argument for the need of differential pricing under PM-JAY and hence, should be used as evidence in the consultative process of reimbursement rate setting.

The analysis also suggests an agenda for future work on the use of cost information to inform these rates and a better understanding of how teaching and city location affect the costs of service provision. In particular, adjustment for patient characteristics is an important future area of work for further refinement of reimbursement rates. Patient-level data from hospitals to determine relative differences in cost of treatment for patients in different case group categories can complement the work presented here on supply-side weights. As evident from the CHSI Study data, the total cost of drugs, consumables, implants and diagnostics explain 57% of the total cost of surgical packages.^5 6^ Moreover, these are the specific resources that vary in terms of quantity among patients with different levels of severity. For the remaining resources including the human resources, capital and overhead, the length of stay is a useful proxy of variation in resource utilisation by levels of severity. This implies that two sets of weights, which differentiate variations between the two sets of input resources, could help to determine a weighted differential reimbursement rate for each case or health benefit package. Accordingly, it is proposed integrating the collection of patient-level data on the quantity of drugs, consumables, diagnostic services, implants and length of stay within PM-JAY’s transaction management system. A pilot to establish such a cost surveillance system has also been initiated by the National Health Authority in five states.^43^

Our study provides new insights into the determinants of the cost of medical and surgical care and provides important evidence for generating price adjustment levels for a differential case-based payment system under India’s PM-JAY. The cost weights suggested under our analysis are necessarily indicative, nonetheless, provide crucial information and should be used along with consultative process. More research is required to determine the base reimbursement rate while improvements to the claims data system to provide patient-level cost data could be used to determine weights for case-mix and severity.

## Supplementary material

10.1136/bmjopen-2023-076155 online supplemental file 1

## Data Availability

Data are available upon reasonable request.
