# Supplementary material for: Refining the provider payment system of India’s government-funded health insurance programme: an econometric analysis
Source: BMJ Open. 2023 Oct 19;13(10):e076155. doi: 10.1136/bmjopen-2023-076155 (PMC10603525; doi:10.1136/bmjopen-2023-076155)
Supplement: online supplemental file 1 [file bmjopen-2023-076155supp001.pdf]

Refining the Provider Payment System of India's Government Funded Health Insurance Program: An Econometric Analysis

Supplementary material

Alternative model – inpatient bed days

Table 1: Results of unit cost function: Inpatient per bed day

Variable	Std. β	UnStd. β	SE β	95% Confidence Interval	
				Lower	Upper
Annual Inpatient admissions	-0.896	-0.669***	0.048	-0.764	-0.575
Number of inpatient beds	0.370	0.313***	0.065	0.184	0.442
Average Length of Stay (ALOS)	-0.441	-0.771***	0.093	-0.956	-0.587
Location (reference: tier 3 city)					
Tier 1	0.067	0.221	0.164	-0.103	0.544
Tier 2	0.154	0.322**	0.109	0.107	0.537
Teaching status (reference: non-teaching hospitals)					
Teaching Hospitals	0.324	0.829***	0.150	0.534	1.124
(Constant)		11.906***	1.147	9.642	14.170
R^2	0.7126				
Adjusted R^2	0.6972				

Note: 1) This model does not include the outliers in capacity utilisation. 2) Further variables included in the model: Doctor paramedics ratio; Specialist paramedics ratio; State GSDP absolute; State Health Index **3)** *p<0.05; **p<0.01; ***p<0.001

$ Unit cost, inpatient beds, average length of stay, bed occupancy and annual admissions are log-transformed.

Alternative model – surgical procedure costs

Table 2: Results of unit cost function: Procedure

Variable	Std. β	UnStd. β	SE β	95% Confidence Interval	
				Lower	Upper
Annual procedures	-0.323	-0.173**	0.054	-0.280	-0.067
Bed occupancy	-0.099	-0.138	0.115	-0.366	0.089
Speciality	0.149	0.558	0.361	-0.158	1.274
Location (reference: tier 3 city)					
Tier 1	0.196	0.522*	0.261	0.004	1.039
Tier 2	0.309	0.593**	0.178	0.241	0.946
Teaching status (reference: non-teaching hospitals)					
Dummy (Teaching)	0.282	0.567*	0.244	0.083	1.051
(Constant)		9.968***	1.812	6.376	13.559
R^2	0.3502				
Adjusted R^2	0.2966				

Note: 1) This model include the outliers in capacity utilisation. 2) Further variables included in the model: Doctor paramedics ratio; Specialist paramedics ratio; State GSDP absolute; Annual OT procedures; State Health Index. **3)** Public and private classification variable not included in cost functions as not interested in weights by "ownership of hospital".**4)** *p<0.05; **p<0.01; ***p<0.001
$ Unit cost, inpatient beds, bed occupancy, annual admissions, state absolute GSDP are log-transformed.
