# Supplementary material for: Refining the provider payment system of India’s government-funded health insurance programme: an econometric analysis
Source: BMJ Open. 2023 Oct 19;13(10):e076155. doi: 10.1136/bmjopen-2023-076155 (PMC10603525; doi:10.1136/bmjopen-2023-076155)
Supplement: Supplementary file 2 [file bmjopen-2023-076155.reviewer_comments.pdf]

PEER REVIEW HISTORY

BMJ Open publishes all reviews undertaken for accepted manuscripts. Reviewers are asked to complete a checklist review form (**http://bmjopen.bmj.com/site/about/resources/checklist.pdf**) and are provided with free text boxes to elaborate on their assessment. These free text comments are reproduced below.

ARTICLE DETAILS

**TITLE (PROVISIONAL)**	Refining the Provider Payment System of India's Government Funded Health Insurance Program: An Econometric Analysis
**AUTHORS**	Prinja, Shankar; Bahuguna, Pankaj; SINGH, MANINDER PAL; GUINNESS, LORNA; Goyal, Aarti; Aggarwal, Vipul

VERSION 1 – REVIEW

**REVIEWER**	Riewpaiboon, Arthorn<br>Mahidol University, Pharmacy
**REVIEW RETURNED**	28-Apr-2022

**GENERAL COMMENTS**	1. Key words: price, reimbursement rate, payment, cost. The study is to explore weights for adjusting the reimbursement rate or payment of the insurance program. The term "price", particularly for private hospitals, is referred to selling prices or charges of medical services. Price of same service can be different for different clients. In the Objectives, it is already mentioned that "Reimbursement rates in national health insurance schemes are frequently weighted to account for differences in the costs of service provision." Be careful, "price" and "cost" are not the same. Actually, it is cost weights as inputs for adjusting differential reimbursement rate or payment of insurance program. I would suggest that the term "price" is not appropriate for this study (whole manuscript).<br>2. The weights in form of percent changes are estimated based on standardized regression coefficients of the fitted regression models. This is a wrong interpretation of standardized regression coefficient. It is "A change of 1 standard deviation in X is associated with a change of β standard deviations in Y" (a sample of sources: https://quantifyinghealth.com/standardized-vs-unstandardized-regression-coefficients/). Actually, it should be based on unstandardized regression coefficients. To explore the weights, category of health facilities should be proposed based on significant predictor variables. Then, based on the unstandardized regression coefficients, weighted average cost is estimated for each category. Finally, we can observe the difference in form of percentage changes among categories of health facilities. (a sample of the method applied: Khaing Oo WW, Riewpaiboon A, Youngkong S, Ko K. How Economic Analysis Increases the Awareness of Clinical Services: A Case of Diabetes Mellitus at a Teaching Hospital in Myanmar. Value Health Reg Issues 2021;29:21-7.)<br>3. In MRA, normally, we present a table of potential predictor variables with average value of each variables (mean/ percent). Table 1 should be modified to be such table. In addition, when there is more than one model with different set of potential predictor variables, they should be specified for each model.

	4. In Table 1, what is the meaning of control variable?<br>5. Normality of dependent variable is a basic assumption of the MRA. In model diagnostics, outlying X observations and outlying Y observations must be also checked and removed. These result in higher power of explanation (adj. R2) Therefore, no need to present base models of unadjusted unit cost and with outlying observations. Anyway, I understand that the term Outliers in this study is not same as the outlying observations. In conclusion, the study must follow the MRA's assumption and model diagnostics.<br>6. Table 2, total number of samples of Tier 1-3 (second column) is not 327, a typo?<br>7. Table 4, constant value must be presented. Location of hospital or City type should be used for Tier, instead of Prices. In the footnote, * is used to indicate p value of coefficient. There are some coefficients in the table without any *.<br>8. Page 15, line 54-59 requires references.<br>9. Check the reference style, for instance, there are both full and abbreviated journal titles.<br>10. Discussion must be modified based on the re-analysis in comment 2.

**REVIEWER**	Sood, Neeraj<br>USC
**REVIEW RETURNED**	13-Jun-2022

**GENERAL COMMENTS**	Overall this is a well written paper on an important topic. The authors clearly justify research methods and also describe results well. I don't have any suggestions for improving the paper. Nice job!

VERSION 1 – AUTHOR RESPONSE

Reviewer 1 comments

1. Key words: price, reimbursement rate, payment, cost. The study is to explore weights for adjusting the reimbursement rate or payment of the insurance program. The term "price", particularly for private hospitals, is referred to selling prices or charges of medical services. Price of same service can be different for different clients. In the Objectives, it is already mentioned that "Reimbursement rates in national health insurance schemes are frequently weighted to account for differences in the costs of service provision." Be careful, "price" and "cost" are not the same. Actually, it is cost weights as inputs for adjusting differential reimbursement rate or payment of insurance program. I would suggest that the term "price" is not appropriate for this study (whole manuscript).

Thank you for identifying these important points about terminology. We have made the following edits to address this issue:

- Where have referred to "Price weights" this has been edited to "price adjustments" or "weights" e.g. Abstract, Introduction – 4th and 5th paragraph, Methods – 1st, 2nd and 4th para, Discussion, 2nd para and 9th para
- Methods, 2nd para, 1st line: changed "Prices" to "Costs and therefore prices"
- Methods, 2nd para, 2nd line- changed "several prices" to "the cost of several inputs and processes"

- Where we have previously used location as a proxy for location based prices (Tier city) we use the term “Location”
- Discussion line 3, edited to: “This unique analysis provides the opportunity to propose a system for determining weights for the reimbursement rates for India’s national…….”

2. The weights in form of percent changes are estimated based on standardized regression coefficients of the fitted regression models. This is a wrong interpretation of standardized regression coefficient. It is "A change of 1 standard deviation in X is associated with a change of β standard deviations in Y" (a sample of sources: https://quantifyinghealth.com/standardized-vs-unstandardized-regression-coefficients/). Actually, it should be based on unstandardized regression coefficients. To explore the weights, category of health facilities should be proposed based on significant predictor variables. Then, based on the unstandardized regression coefficients, weighted average cost is estimated for each category. Finally, we can observe the difference in form of percentage changes among categories of health facilities. (a sample of the method applied: Khaing Oo WW, Riewpaiboon A, Youngkong S, Ko K. How Economic Analysis Increases the Awareness of Clinical Services: A Case of Diabetes Mellitus at a Teaching Hospital in Myanmar. Value Health Reg Issues 2021;29:21-7.)
   Thank you for the valuable suggestion. We have revised our analysis based on reviewer’s suggestion and now we present the reimbursement rate weights (or adjustment factors) based on unstandardized regression coefficients. The revised results are presented in Table 3 and 4 in main manuscript; and in the supplementary section. We acknowledge the revised analysis has improved significantly as per comments from the reviewer.

3. In MRA, normally, we present a table of potential predictor variables with average value of each variables (mean/ percent). Table 1 should be modified to be such table. In addition, when there is more than one model with different set of potential predictor variables, they should be specified for each model.
   Thank you for this suggestion. We have added the mean/ percentage values to table 1 as per your guidance, where appropriate.

4. In Table 1, what is the meaning of control variable?
   These are the variables that are important drivers of cost and are considered as part of the cost function but would not be appropriate variables for price adjustments. We acknowledge that this was not clear and so we have deleted the term “Control”, starred these variables and added a table note to explain this.
   “** Variable not considered for price adjustments as impractical or not considered appropriate for compensation (provides the wrong kind of incentive) but are likely to be a key determinant of costs that needs to be accounted for in the model”

5. Normality of the dependent variable is a basic assumption of the MRA. In model diagnostics, outlying X observations and outlying Y observations must be also checked and removed. These result in higher power of explanation (adj. R2) Therefore, no need to present base models of unadjusted unit cost and with outlying observations. Anyway, I understand that the term Outliers in this study is not same as the outlying observations. In conclusion, the study must follow the MRA's assumption and model diagnostics.
   Thank you for this helpful recommendation on presentation of the diagnostics. We have moved the results of the base, unadjusted and poorly performing models to the supplementary material and now only present the final model along with the criteria of selection for the best fit model.

6. Table 2, total number of samples of Tier 1-3 (second column) is not 327, a typo?
   Thank you for spotting this typo. With the changes to table 1, table 2 became redundant and therefore we have deleted this table.

7. Table 4, constant value must be presented. Location of hospital or City type should be used for Tier, instead of Prices. In the footnote, * is used to indicate p value of coefficient. There are some coefficients in the table without any *.
   Thank you for these recommendations. The table 3 and 4 have been revised based on recommendations. We now only present the final model and have put the alternatives in the supplementary material. Prices has been changed to "Location". For the final model in both cases, the insignificant (no star) coefficient is related to one of a set of dummies.

8. Page 15, line 54-59 requires references.
   Thank you for pointing out this omission. We have now added the relevant references.

9. Check the reference style, for instance, there are both full and abbreviated journal titles.
   We have checked this and addressed the reference style where there were inconsistencies

10. Discussion must be modified based on the re-analysis in comment 2.
   We have revised the discussion as suggested in view of comment 2. In addition, more limitations have been added in the discussion section on page 14, lines 22-33 and page 15, lines 1-6. Now, we present the weights generated through this analysis as indicative, which should be used along with expert consultations.

VERSION 2 – REVIEW

**REVIEWER**	Riewpaiboon, Arthorn<br>Mahidol University, Pharmacy
**REVIEW RETURNED**	23-Aug-2023

**GENERAL COMMENTS**	Estimated effect of predictor variables in form of percentage by simply converting unstandardized co-efficient is wrong (See sample given in the previous comment). Actually, the comment has been provided, the authors should explain why do not agree to the reviewer's comment.

VERSION 2 – AUTHOR RESPONSE

Reviewer comments 1

Prof. Arthorn Riewpaiboon, Mahidol University

Comments to the Author: Estimated effect of predictor variables in form of percentage by simply converting unstandardized co-efficient is wrong (See sample given in the previous comment). Actually, the comment has been provided, the authors should explain why do not agree to the reviewer's comment.

Response: We would like to firstly acknowledge the significantly useful inputs provided by the reviewer during the first revision as well as during this second review. During first revision, we had made extensive revisions in the analysis and estimated unstandardized coefficients, which as rightly pointed out by the reviewer, was more appropriate. We have now revised our interpretation of the

unstandardized coefficients, and have analyzed it according to standard recommended methodology which is appropriate when multiple linear regression is undertaken in a log-log fashion, i.e., the dependent variable (unit cost) and independent variables (annual inpatient admissions, number of inpatients beds and ALOS) were log transformed. The formula used for interpretation of estimated effect of predictor variables is $(e^{\beta}-1)\times 100$ %.*

The suitable modifications in the revised manuscript have been made in

Abstract section on page number 2, lines 31-36.

Result section of main manuscript, on page number 11, lines 4-7.

Result section of main manuscript, on page number 12, lines 9-12.

Table 3 and Table 4 in the main manuscript.

Table 1 and Table 2, in the supplementary material.

* Halvorsen R, Palmquist R. The interpretation of dummy variables in semilogarithmic equations. American Economic Review. 1980; 70 (3):474-475.
